# Investigation of Interfacial Layer for Ultrasonic Spot Welded Aluminum to Copper Joints

**DOI:** 10.1038/s41598-017-12164-2

**Published:** 2017-10-02

**Authors:** Zijiao Zhang, Kaifeng Wang, Jingjing Li, Qian Yu, Wayne Cai

**Affiliations:** 1 Department of Materials Science and Engineering, Zhejiang University, Hangzhou, 310027 China; 2 Department of Industrial and Manufacturing Engineering, Pennsylvania State University, University Park, PA 16802 USA; 3 General Motors Global Research and Development Center, Warren, MI 48090 USA

## Abstract

The bonding formation for ultrasonic welding of dissimilar metals has been shrouded in mystery because of the complex thermomechanical behavior at the bonding interface. We investigated the microstructure and phases at the bonding interface of ultrasonically welded aluminum to copper joints using transmission electron microscopy, and found a ~10 nm thick transition layer composed of amorphous phase and nanocrystallines, which was believed to form the bonding between these two metals in addition to mechanical interlocking observed at a larger scale. Interdiffusion of parent elements (i.e. Al and Cu) was noticed in the amorphous phase, which was mainly driven by plastic deformation in solid state introduced by ultrasonic vibration. High densities of dislocations and stacking faults were also observed in the parent metals close to the transition layer, confirming the effects of severe plastic deformation.

## Introduction

Multi-material structures are of increasing demand to improve product performance and satisfy functional needs. Matinsen *et al*.^1^ classified the joining technologies for dissimilar materials according to joint formation mechanisms, i.e. mechanical, chemical, thermal (including fusion and solid-state), or hybrid processes. Wherein, ultrasonic metal welding^2^, as a solid-state process, becomes popular in electronic, automotive, and aerospace for its low heat generation, and fast and easy automation. Especially in recent years, there is a rapid growth of ultrasonic welding for joining thin, multiple, and highly conductive dissimilar materials. One driving force behind this is the increasing application of lithium-ion battery in consumer electronics, electric vehicles, and smart grids, where ultrasonic welding is a dominant joining method to assemble the cell terminals and bus bars, and the targeted metals are usually Al, Cu, and other high thermal conductivity materials. Existing investigation of Al-Cu joining focused primarily on the process optimization. Although it is known that heating source comes from the interfacial friction between the jointed materials, the physics behind bonding mechanism is still unclear.

Understanding the bonding mechanisms for dissimilar materials has received increasing interests for ultrasonic welding. Yang *et al*.^3^ addressed that there were several bonding formations during ultrasonic welding, i.e. mechanical interlocking, metal melting, and diffusion. Xu *et al*.^4^ investigated the microstructure of steel/Al joints, and found that intermetallic compounds (IMCs) existed due to the high interfacial temperature (higher than the melting temperature of applied aluminum alloys). Ren *et al*.^5^ found banded grain-refinement in Mg alloy to Ti alloy joints, where neither diffusion layer nor IMCs were observed. Lu *et al*.^6^ and Fujii *et al*.^7^ presented that dynamic recrystallization might occur concurrently during ultrasonic welding. The bonding mechanism of Al-Cu ultrasonic welding has been investigated by a number of researchers, and reported that the microstructure of interfacial layer for Al-Cu joints varies with different processing conditions. For instance, Yang *et al*.^8^ found an IMC layer composed of CuAl_2_ and Cu_9_Al_4_ in ultrasonically welded AA6061-Cu joints; Zhao *et al*.^9^ investigated the effect of ultrasonic welding energy on interfacial microstructure of Al-Cu joints during ultrasonic welding, and reported that Cu_9_Al_4_ intermetallic was more likely to occur at a high welding energy; Lee and Kwon^10^ investigated the diffusion bonding between Al and Cu during vacuum hot pressing, and the HRTEM images revealed that three IMC layers were generated during the diffusion bonding process. In general, the bonding formations could be specific to certain material combinations or processing conditions.

In addition to ultrasonic welding, another high strain-rate joining method (i.e. explosive welding) has received attentions for bonding mechanism investigation. Amorphization was reported at the bonding interface of explosively welded joints^11–13^, which is believed to be the result of rapid solidification from liquid phase when melting temperature reached. It should be noticed that the completion time for explosive welding is usually in microseconds, introducing rapid heating and solidification for the work material system; whereas the process of ultrasonic welding takes around one second. It is necessary to include the process condition in this study although a similar amorphous phase could be found from post-process analysis.

## Results and Discussion

**Figure 1:**
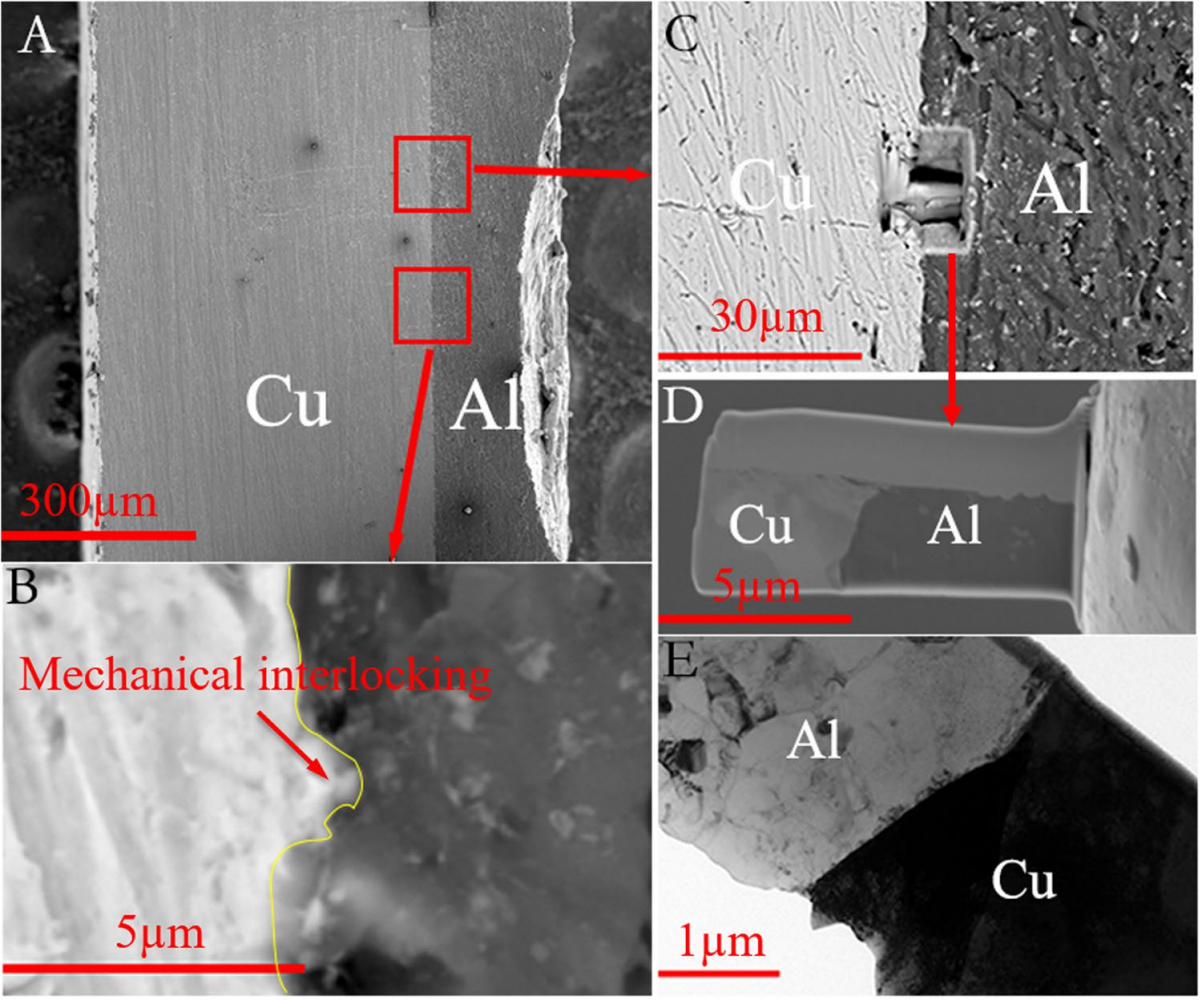
(**A**) overview of the weld cross-section, (**B**) high-magnification BSE image for mechanical interlocking, (**C**) site selection for FIB specimen, (**D**) prepared FIB specimen for TEM, and (**E**) low-magnification cross-sectional TEM image of Al-Cu interface.

In this study, the as-received materials are pure aluminum (AA1100) and pure copper (C110), both are in annealed condition. An overview of the weld cross-section between Al and Cu is shown in Fig. 1A, where the welding line (i.e. bonding interface) is of interest. To unveil the characteristics of the bonding interface in detail, the specimen was cut and extracted from weld region by focused ion beam (FIB, Dual-beam FIB system FEI Quanta 3D FEG), as marked in Fig. 1A; and the back scattered electron (BSE) image of the cut region is presented in Fig. 1C. The lifted and thinned FIB sample is shown in Fig. 1D, in which the Al-Cu interface is clearly observed. Based on the diffraction contrast produced under the bright-field observation, low-magnification cross-sectional transmission electron microscopy (TEM) image illustrates the structure of parent Al, Cu and the Al-Cu interface (Fig. 1E). It is noted that the welding line is not as flat as the one shown under low-magnification (Fig. 1A). From the relatively high-magnification BSE image (Fig. 1B), wavy welding line is observed as a result of the material flowing across the interface^14^, defined as mechanical interlocking^15^. In addition, there are no apparent transition zones at both parent Al and Cu sides under low-magnification in Fig. 1.

**Figure 2:**
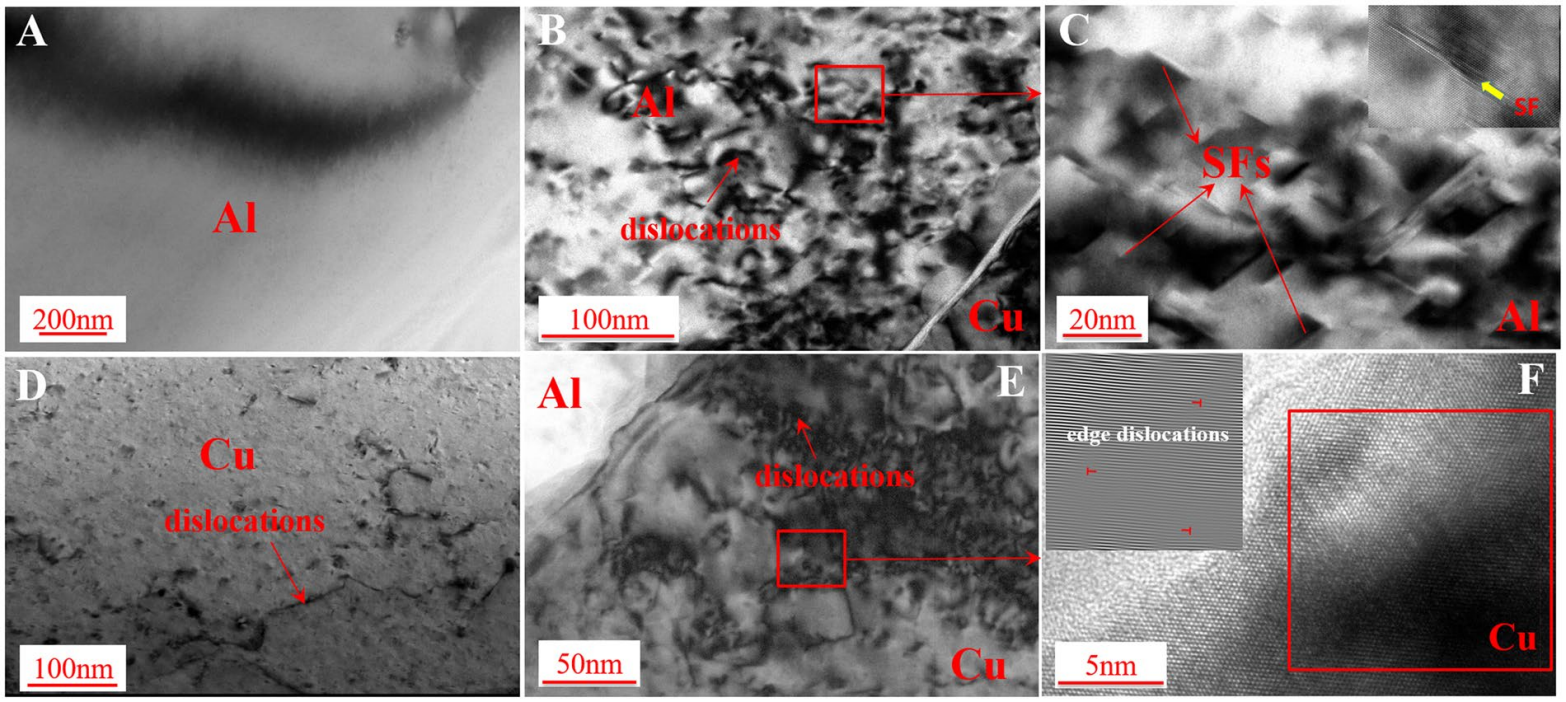
(**A**) TEM image of Al before welding, (**B**) dislocations in Al grains near interface after welding, (**C**) SFs in Al grains near interface, (**D**) TEM image of Cu before welding, (**E**) dislocations in Cu grains near interface after welding, and (**F**) edge dislocations in Cu grains near interface.

Figure 2 summarizes the structures of parent Al and Cu grains near the interface observed under the TEM two-beam condition. The structures of as-received Al and Cu before welding (Fig. 2 A and D) are also shown to compare the microstructure changes. In this study, the as-received materials are in annealed condition. The density of dislocations in Cu sheet (Fig. 2D) is low before welding and is even lower in aluminum sheet (Fig. 2A), which indicates that the annealing process entirely (or partially) recovered the cold work from the sheet fabrication (rolling). After welding, large amounts of dislocations were observed in both Al and Cu grains near the interface as indicated in Fig. 2 B and E, respectively. Although it is known that Al has a high stacking-fault energy^16^, high density stacking faults (SFs) are observed in Al grains, as illustrated by the low-magnification bright-field TEM image and its inset high-resolution transmission electron microscopy (HRTEM) image (Fig. 2C), which usually appear during severe plastic deformation. The HRTEM image (Fig. 2F) and the 1D fast Fourier transform (FFT) pattern (inset of Fig. 2F) demonstrate that high density of 1/2<110> edge dislocations are prevalent along {111} slip planes within the face centered cubic (FCC) Cu grain, indicating that dislocations were nucleated and glided along the FCC slip systems to accommodate the plastic deformation during the welding process. All these observations confirm that severe plastic deformation occurred in ultrasonic welding, which agree with the results proposed by Koike^17^ and Szlufarska *et al*.^18^, i.e. an increasing amount of dislocations will be activated and accumulated around the interface as plastic strain increases. In addition, the large amounts of dislocations and SFs within grains resulting from the plastic deformation imply that there is no significant grain recrystallization near the interface. These phenomena indicate that the welding temperature for Al-Cu is not high enough at the chosen location to form IMCs under this welding condition, unlike previous reports on ultrasonically welded Al-Cu joints using a higher welding energy^8,9^.

**Figure 3:**
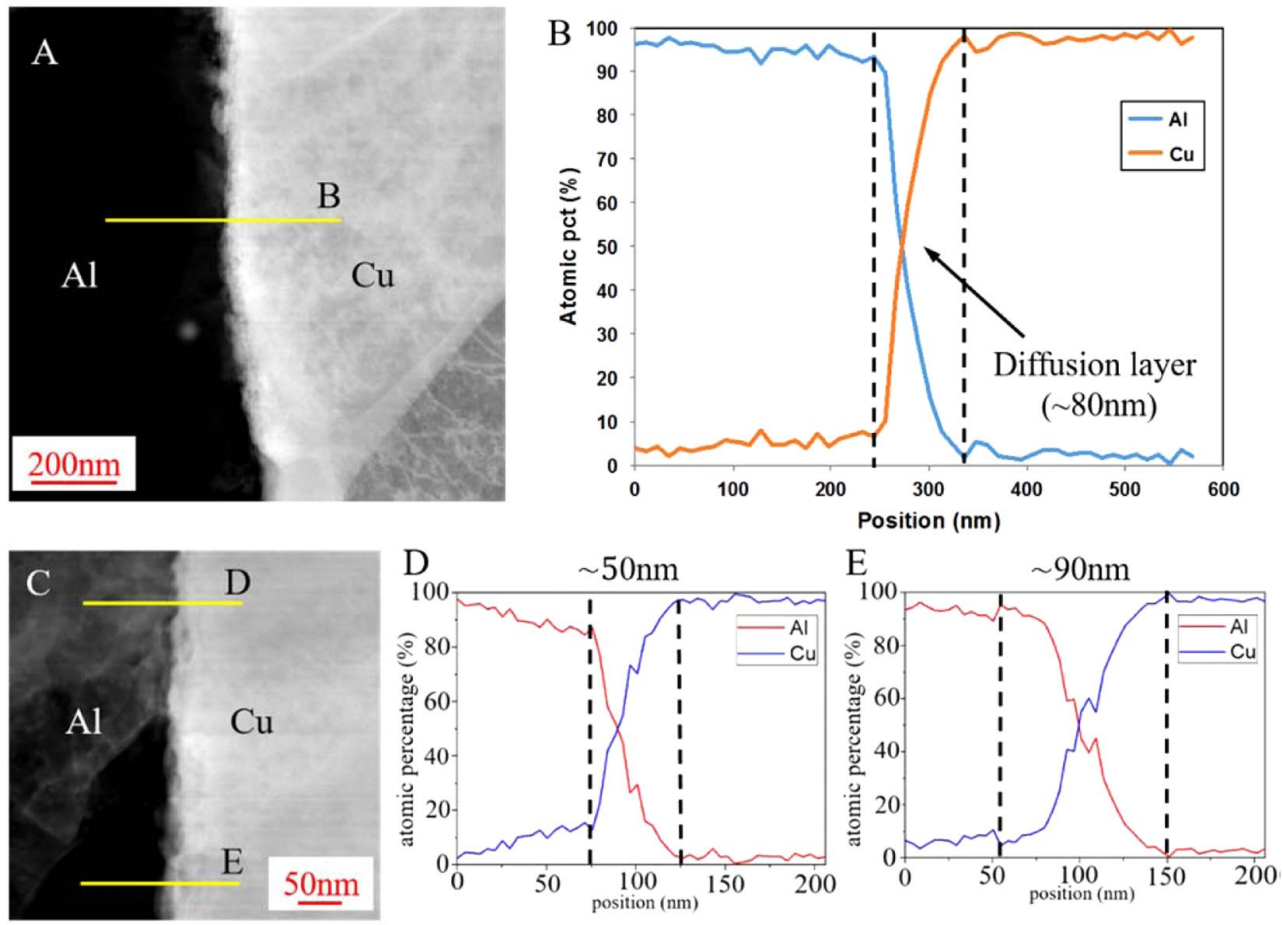
(**A**) STEM image around the weld interface, (**B**) the element distributions obtained by EDXS, (**C**) high-magnification STEM image around the weld interface, (**D**) and (**E**) the element distributions in different welded region with a high-magnification.

To identify the bonding formation at the interface, energy-dispersive X-ray spectroscopy (EDXS) in scanning transmission electron microscopy (STEM) has been used for the detection of chemical composition around the Al-Cu interface. Figure 3A,B show the STEM image around the bonding interface and the corresponding element distribution along the yellow line perpendicular to the interface, respectively. From the EDXS line scanning (Fig. 3B), it can be seen that there is a thin diffusion layer (~80 nm) between Al and Cu, where the element distributions of Al and Cu have contrary tendency along the scanning line, as shown in Fig. 3B. To ensure the thickness of diffusion layer, more line scanning measurements were conducted; and Fig. 3D,E present the corresponding element distributions along the yellow lines in Fig. 3C. It is found that the thickness of the diffusion layer is not constant (~50 nm in Fig. 3D and ~90 nm in Fig. 3E), indicating a non-uniform welding diffusion area along the welding line.

**Figure 4:**
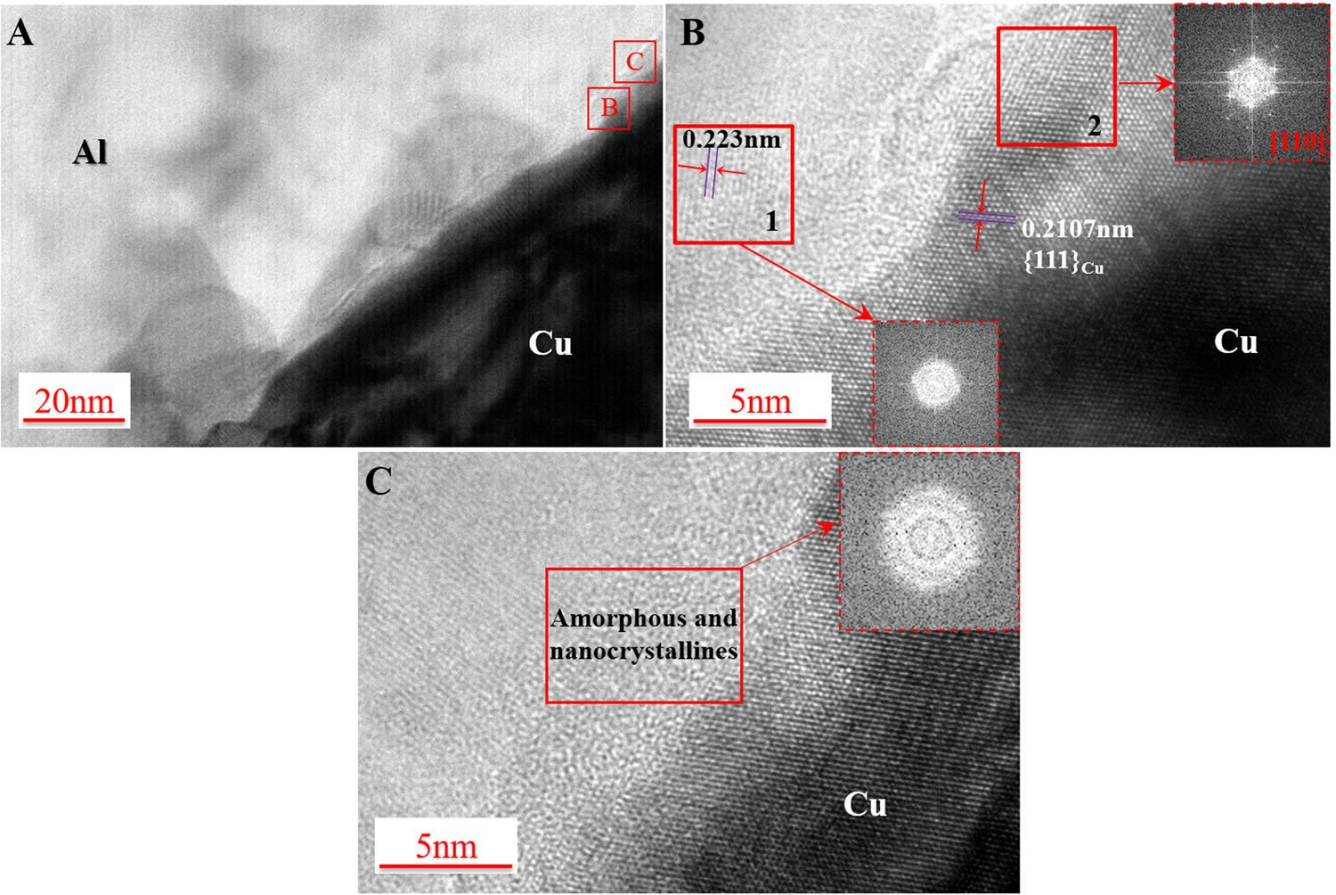
(**A**) Low magnification bright-field image of the diffusion layer, (**B**) and (**C**) HRTEM images of the transition layer taken from (**A**).

The structure of the diffusion layer was investigated in nano-scale through the analyses of both TEM and HRTEM images. The TEM specimen was tilted to align one of the Cu grains near the interface to be parallel to the <110> zone axis. The low magnification bright-field image is illustrated in Fig. 4A, where an apparent thin transition layer is found in the diffusion layer. In the transition layer with a width of ~10 nm, amorphous phase and nanocrystalline are observed from HRTEM images (Fig. 4B,C), which can be confirmed by FFT patterns with the characteristics of diffuse halo and polycrystalline taken from the corresponding areas marked by red boxes. Also, in the nanograins, the spacing of partially intact lattice planes for Cu was measured to be 0.2107 nm, which is slightly larger than that of pure Cu (0.2088 nm). Similarly, the measured spacing of Al (0.2230 nm) is larger than that of pure Al (0.2024 nm). Thus, the nanocrystallines are speculated as Cu solid solutions with Al solutes near pure Cu, and Al solid solutions with Cu solutes close to Al.

It is known that temperature evolution plays an important role in the bond interfacial phase generation. For explosive welding, the generation of amorphous phase occurs due to the rapid solidification of molten material^11^. For ultrasonic metal welding process, real time temperature monitoring was conducted by Zhao *et al*.^19^ with a similar setup condition as this study. In their study, the maximum welding temperature can rise up to 377 °C, which is much lower than the eutectic temperature (548.2 °C) in the Al-Cu equilibrium phase diagram. It indicates that local melting is not likely to occur during the welding condition studied. The estimation of the EDXS results (Fig. 3) together with the atomic lattice in the transition layer in the HRTEM images (Fig. 4) suggests that the bonding between Al and Cu takes place by the enhanced interdiffusion of the parent elements during the severe plastic deformation at a high strain rate.

Besides extremely rapid cooling, several researches reported that solid-state amorphous phase could be generated after severe plastic deformation^20–22^, with the process of amorphous formation as follows: (1) generation of dislocations within grains; (2) formation of fragmented and then ultra-fine grained structures; and (3) appearance and spread of amorphous structures. It is noted that individual nano-sized crystalline grains could be found in the amorphizing structure and their grain number and size will decrease as strain increases, until completely diminish^22^. Sagel *et al*.^23^ reported that grain refinement as a type of structural disordering is usually seen in alloys under severe plastic deformation and occurs prior to the onset of amorphization. To accommodate the plastic deformation, introduced grain boundaries could raise the material structural system to energy state above the amorphous state to drive the amorphization^24^. Another factor is the dislocation accumulation which contributes to the atomic disorder, lattice strain, and eventually the collapse of crystalline structure for amorphization^25^. For the multilayer ultrasonic welding process, Lee *et al*.^26^ reported that there was a relative displacement between adjacent welding layers, resulting in interfacial friction. In this study, under both interfacial friction and the high welding pressure, plastic deformation occurred near the interface, which leads to higher density of dislocations in both Al and Cu grains compared to the un-welded materials, as shown in Fig. 2. During the further welding process, more dislocations are accumulated at the interface, resulting in the grain refinement and simultaneously raising the energy state to drive amorphization, as shown in Fig. 4.

In summary, with the aid of advanced interfacial structure characterization and analysis, it is revealed that severe plastic deformation contributes to the formation of transition layer composed of nanocrystallines and amorphous phase, as well as high densities of dislocations and SFs in parent Al and Cu. It is believed the local melting is not likely to happen for the current process. A couple of bonding formations, including enhanced interdiffusion introduced in amorphous phase and mechanical interlocking, are observed in the current Al-Cu joint, together contributing to the bonding strength. These results lead to a significant improvement on the understanding of ultrasonic welding bonding mechanism for dissimilar metals as well as the general solid-state welding involving thermomechanical principles.

## Methods

**Figure 5:**
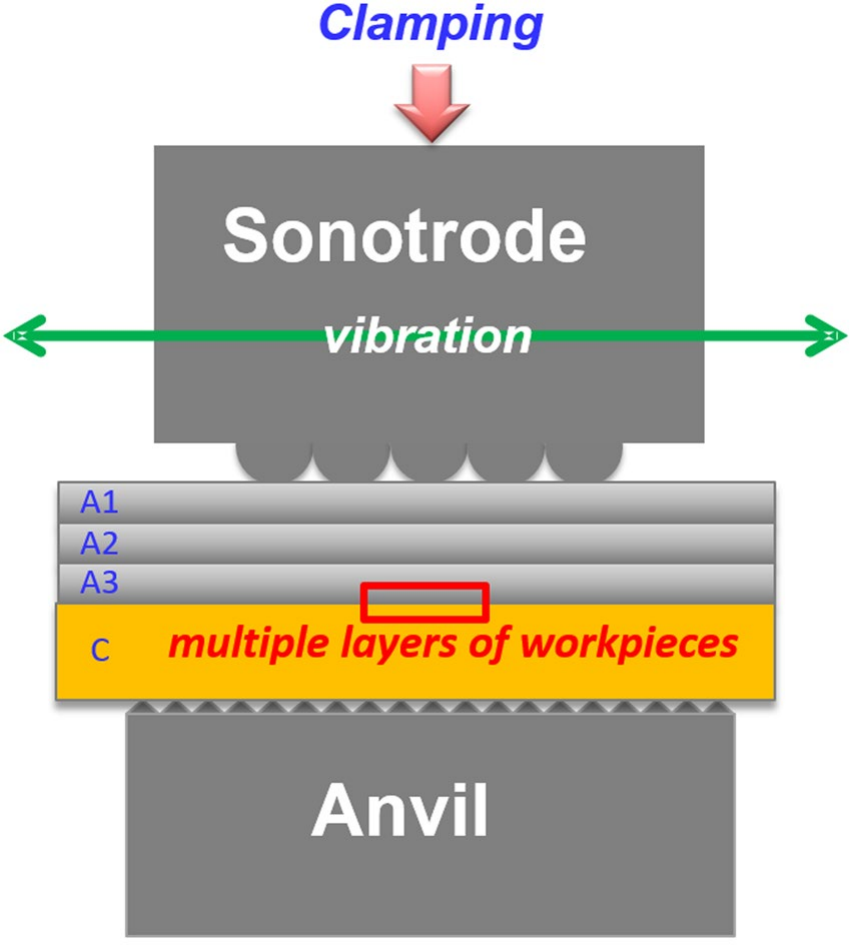
Illustration of ultrasonic welding setup and material layout.

Ultrasonic welds were produced by lapping three layers of pure Al sheet (45 mm × 19 mm × 0.2 mm) over a layer of pure Cu sheet (45 mm × 19 mm × 0.5 mm). The setup of the machine with material layout is illustrated in Fig. 5. A Branson® L20 with a 20 kHz ultrasonic welder was used in this study. The nominal welding pad on the sonotrode was 12.7 mm × 8 mm including 5 rows and 3 columns of spherical knurls with a radius of 1.2 mm; and the vibration direction was along the longitudinal direction of the welding pad^27^. Before welding, a clamping force was applied sequentially to fix the overlapped Al and Cu sheets. The qualities of the welds can be controlled by varying three basic parameters in this close-loop ultrasonic welding system, i.e., welding energy, welding pressure, and welding amplitude. Based on our previous investigation^15^, welding parameters of welding energy 500 J, welding pressure 30 psi, and welding amplitude 35 µm, were used to generate a good quality weld in this study. To characterize the bonding interface, several techniques were applied, including TEM, STEM, HRTEM, and EDXS. The site selection for FIB specimen is under a spherical knurl, where the local welding pressure is higher compared to other positions leading to a larger deformation and heat generation. Future work will include characterization of the microstructures at different locations at all welding interfaces.
